# Relationship between Occupational Exposure to Airborne Nanoparticles, Nanoparticle Lung Burden and Lung Diseases

**DOI:** 10.3390/toxics9090204

**Published:** 2021-08-30

**Authors:** Valérie Forest, Jérémie Pourchez, Carole Pélissier, Sabyne Audignon Durand, Jean-Michel Vergnon, Luc Fontana

**Affiliations:** 1 Centre CIS, Mines Saint-Etienne, Univ Lyon, Univ Jean Monnet, INSERM, U1059 Sainbiose, F-42023 Saint-Etienne, France; pourchez@emse.fr; 2 Department of Occupational Medicine, University Hospital of Saint-Etienne, F-42055 Saint-Etienne, France; carole.pelissier@chu-st-etienne.fr (C.P.); luc.fontana@chu-st-etienne.fr (L.F.); 3 Univ Lyon, Univ Eiffel, Univ Lyon 1, Univ St Etienne, IFSTTAR, UMRESTTE, UMR_T9405, F-42005 Saint-Etienne, France; 4 EPICENE Team, Inserm U1219, Bordeaux Population Health Research Center, University of Bordeaux, F-33076 Bordeaux, France; sabyne.audignon@u-bordeaux.fr; 5 Department of Occupational and Environmental Medicine, Bordeaux Hospital, F-33400 Talence, France; 6 Univ Lyon, Univ Jean Monnet, INSERM, U1059 Sainbiose, F-42023 Saint-Etienne, France; Jean.Michel.Vergnon@univ-st-etienne.fr; 7 Department of Chest Diseases and Thoracic Oncology, University Hospital of Saint-Etienne, F-42055 Saint-Etienne, France

**Keywords:** biomonitoring, nanoparticles, lung diseases, mineralogical analysis of broncho-alveolar lavages, occupational exposure

## Abstract

The biomonitoring of nanoparticles in patients’ broncho-alveolar lavages (BAL) could allow getting insights into the role of inhaled biopersistent nanoparticles in the etiology/development of some respiratory diseases. Our objective was to investigate the relationship between the biomonitoring of nanoparticles in BAL, interstitial lung diseases and occupational exposure to these particles released unintentionally. We analyzed data from a cohort of 100 patients suffering from lung diseases (NanoPI clinical trial, ClinicalTrials.gov Identifier: NCT02549248) and observed that most of the patients showed a high probability of exposure to airborne unintentionally released nanoparticles (>50%), suggesting a potential role of inhaled nanoparticles in lung physiopathology. Depending on the respiratory disease, the amount of patients likely exposed to unintentionally released nanoparticles was variable (e.g., from 88% for idiopathic pulmonary fibrosis to 54% for sarcoidosis). These findings are consistent with the previously performed mineralogical analyses of BAL samples that suggested (i) a role of titanium nanoparticles in idiopathic pulmonary fibrosis and (ii) a contribution of silica submicron particles to sarcoidosis. Further investigations are necessary to draw firm conclusions but these first results strengthen the array of presumptions on the contribution of some inhaled particles (from nano to submicron size) to some idiopathic lung diseases.

## 1. Introduction

Nanoparticles are ubiquitous in nature, naturally occurring as by-products of wild fires, volcanic eruptions, and other natural processes, and are usually called ultra-fine particles (UFP). Nanoparticles can also be a result of human activities unintentionally produced and present in polluting emissions, such as welding fumes, cigarette smoke, aircraft waste gas, or diesel exhaust, also called UFP. In addition to these sources, a number of artificial nanoparticles, engineered nanomaterials (NM) which exhibit unique physical, chemical and/or biological characteristics associated with their nanostructure, have been developed and produced in a controlled, engineered manner to exploit their novel properties and functions. Due to the tremendous development of nanotechnologies during the last few decades and the subsequent potential exposure of humans to nanomaterials, nanotoxicology is a rapidly evolving research field. According to Stone et al. in a review [1], UFP and NM toxicology are not two distinct fields. Rather, they overlap extensively with the potential to extrapolate from one to the other in many respects. Furthermore, for these authors, ambient particulate matter research provided evidence of potential health impacts for UFPs, and NM toxicology has largely provided essential evidence of the mechanistic plausibility of these health effects. Finally, according to Stone et al., it seems safe to conclude that UFPs and NMs share the same general biological mechanisms of adverse effects.

In their review, Manno et al. defined biomonitoring as “the repeated, controlled measurement of chemical or biological markers in fluids, tissues, or other accessible samples from subjects exposed or exposed in the past or to be exposed to chemical, physical or biological risk factors in the workplace and/or the general environment” [2]. Consequently, in a context of health risk assessment, biomonitoring can be a particularly useful approach. Biomarkers used in human health studies typically fall within three categories: biomarkers of exposure, effect, and susceptibility [3]. They can bring critical information on the relationship between exposure to a harmful substance and biological/pathological effects.

Biomonitoring has been widely used in pulmonology, especially in the case of pneumoconiosis. One typical example is the assessment of asbestosis bodies in patient lung tissues or in broncho-alveolar lavage (BAL) fluids which has allowed defining values specific of diseases [4,5,6]. More recently, it has been suggested that the chemical composition of BAL from idiopathic pulmonary fibrosis patients had a specific profile that can be distinguished from that of patients with other interstitial lung diseases or healthy subjects [7]. The extension of this approach to the nanotoxicology field, although it has to face some technical challenges [8,9], could be very interesting especially to get new insights into the role of inhaled biopersistent nanoparticles in the etiology or development of some respiratory diseases. Indeed, although the impact of air pollution, including the contribution of nano-sized particles, on human health has been well documented [10], fewer data are available on the effects of nanoparticles, either engineered or unintentionally released, in the context of occupational exposure.

**Figure 1:**
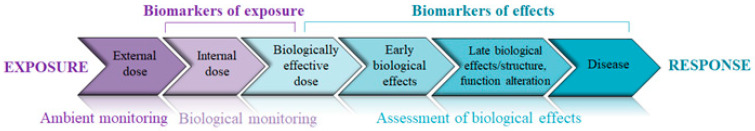
The mineralogical analysis of metal load extracted from pulmonary fluids could be used as an indicator of exposure to nanoparticles and could contribute to the assessment of potential causal links between the presence of inhaled biopersistent nanoparticles in the lungs and respiratory diseases.

The biological monitoring of nanoparticles in human lung tissues or fluids could fill a gap and represents a promising way to investigate potential causal links between an exposure to inhaled nanoparticles and biological effects and even diseases [8,11,12,13,14] (Figure 1).

Indeed, mineralogical analyses of BAL (i.e., biological monitoring of inhaled particles) allow quantifying the internal dose of inhaled biopersistent nanoparticles in a lung sample, which differs from the external dose that can be measured by ambient monitoring (i.e., atmospheric metrology). The assessment of the internal dose is a first step towards the characterization of persistent nanoparticles in tissues and the understanding of this potential source of adverse effects.

We adopted this approach to detect and quantify nanoparticles in various types of clinical samples. We developed optimized protocols for each kind of biological matrix to isolate the micro, sub-micro, and nano fractions of various types of inorganic particles and thus perform comprehensive mineralogical analyses. We were thus able to determine the nanoparticle load in patients’ biological samples such as seminal and follicular fluids [15], colon [16], amniotic fluids [17], or BAL [18,19,20]. We especially focused our attention on these latter, as the biomonitoring of biopersitent nanoparticles in the lung could be particularly relevant in the case of respiratory diseases. Indeed, the respiratory tract represents the main route of entry for nanoparticles in the body and despite the lack of clear evidence it has been suggested that inhaled engineered nanoparticles accumulated in the lungs could be responsible, or at least could contribute, to idiopathic respiratory diseases.

We previously conducted a clinical trial on a cohort of 100 patients (NanoPI clinical trial, ClinicalTrials.gov Identifier: NCT02549248). We separated micron-sized particles (>1 µm) from submicron (100 nm–1 µm) and nano-sized particles (<100 nm) contained in BAL from patients who suffered from interstitial lung diseases (ILD). We then determined the metal load in each of these size-fractions. We evidenced a concentration of submicron silica particles higher in patients suffering from sarcoidosis than in patients suffering from other ILD, suggesting a potential role of these inhaled particles in the etiology and/or development of sarcoidosis [19]. Similarly, we observed a concentration of titanium nanoparticles higher in patients suffering from idiopathic fibrosis than in patients suffering from other ILD allowing to suspect a relationship between titanium nanoparticles and idiopathic pulmonary fibrosis even though in this case we had a too limited number of patients to reach a satisfactory statistical power to draw firm conclusions.

To complement mineralogical analyses of BAL and offer a comprehensive vision of the events from exposure to airborne nanoparticles to the biological response induced (Figure 1), we investigated associations between respiratory diseases and occupational exposures. To that purpose, we estimated the exposure to inhaled unintentionally released nanoparticles of the patients for each job held in their working life.

Thus, the objective of the present paper was to further investigate the relationship between the biological monitoring of nanoparticles in human BAL, interstitial lung diseases, and occupational exposure using a retrospective occupational exposure to unintentionally released nanoparticles assessment. Getting a complete picture from exposure to disease illustrates a comprehensive and useful approach in terms of human health risk assessment.

## 2. Materials and Methods

### 2.1. Patients

A prospective, monocentric and exploratory study called NanoPI (ClinicalTrials.gov Identifier: NCT02549248) was carried out during two years at the University Hospital of Saint-Etienne (Chest diseases and thoracic oncology Department). One-hundred patients exhibiting a clinical image of diffuse ILD and in need of a bronchoscopy associated with a BAL were included in this study after being fully informed and having given their written consent. Our protocol was in accordance with ethical principles defined by the World Medical Association declaration of Helsinki and subsequent amendments and was approved by an ethics committee (Comité de Protection des Personnes, Sud-Est I) as well as by the French agency regulating biomedical research (Agence Nationale de Sécurité du Médicament et des produits de santé, ANSM). 

The following criteria of inclusion were applied: (i) patients with an ILD determined based on clinical signs and CT scan, requiring a flexible bronchoscopy associated with a BAL; (ii) patients older than 18; (iii) patients who had given their voluntary, informed and written consent; (iv) patients having a social insurance or beneficiary (mandatory for any French clinical study). Patients were excluded in the following cases: (i) patients who had not given their consent; (ii) when flexible bronchoscopy or BAL was not possible; (iii) patients under legal protection or pregnant women; (iv) patients with contagious disease (e.g., HIV infection, tuberculosis, viral hepatitis) for safety reasons.

### 2.2. Broncho-Alveolar Lavages

BAL were performed by injecting 50 mL of a warmed saline solution in the selected area of the patient lung. This solution was slowly aspirated, the collected sample constituting the bronchial wash (BW). Then, 2 to 4 additional 50 mL doses of warmed saline solution were injected and aspirated successively, the collected sample representing the BAL strictly speaking. After cytological analysis of the samples performed by the Histology-Cytology Department of the University Hospital of Saint-Etienne, the remaining samples (5 mL of BW and 20 mL of BAL) were added with an equivalent volume of sodium hypochlorite and stored at 4 °C until the mineralogical analyses were performed. 

### 2.3. Sample Pre-Treatment and Analysis

We previously developed and validated a size fractionation protocol allowing to separate microparticles (>1 µm) from submicron particles (ranging from 100 nm to 1 µm) and nanoparticles and ions (particles < 100 nm), described extensively in our previous publications [18,19,20]. We applied this protocol to BAL and BW samples. Dynamic light scattering (DLS) method (Nanozetasizer®, Malvern Instrument, Orsay, France) allowed verifying the efficiency of the size fractionation. All fractions were also analyzed using inductively coupled plasma atomic emission spectroscopy (ICP-AES, Jobin-Yvon JY138 Ultrace) to assess for each metal the quantity of matter expressed in parts-per-billion (ppb), i.e., as ng/mL. The content of BAL and BW in aluminum (Al), beryllium (Be), cobalt (Co), chromium (Cr), copper (Cu), iron (Fe), nickel (Ni), silicon (Si), titanium (Ti), tungsten (W), zinc (Zn), and zirconium (Zr) was thus determined. “Blank” samples were also included to ensure samples were not contaminated with particles present in the environment, the materials, and solutions used. These control samples consisted of saline solution aspirated through the bronchoscope and that underwent exactly the same processes as clinical samples did. 

### 2.4. Comparison to Clinical Data

More than 200 different conditions are grouped under the term interstitial lung disease, classified together because of similar clinical, radiographic, physiologic, or pathologic manifestations [21]. These diseases can be subdivided into those with a known origin (e.g., systemic disease, iatrogenic causes by drug, radiation, extrinsic allergic, pneumoconiosis, post-infectious) and those without, the latter usually called idiopathic interstitial pneumonias (mainly sarcoidosis, other granulomatous ILD and idiopathic pulmonary fibrosis). Patients from our cohort were thus first classified in two groups depending on the origin of the disease they suffer from, either with a known etiology or idiopathic. Details on the cohort are reported in Table 1. We then focused on sarcoidosis and idiopathic pulmonary fibrosis, two groups for which we suspected the contribution of Si submicron particles and Ti nanoparticles, respectively [19].

### 2.5. Retrospective Occupational Exposure to Nanoparticles Assessment

Patients were asked about all occupations held and industrial sectors, for at least six months since leaving school during their working life, up to the date of the diagnosis. For each occupation, the employer’s sector was coded into the French classification of activities (NAF, Nomenclature d’Activités Françaises, 1999) of the National Institute for Statistics and Economics Studies (INSEE) [22], and the occupation was coded according to the International Standard Classification of Occupations (ISCO, 1968) of the International Labour Organisation, The International Labour Office [23].

Patients were also asked about working conditions such as exposure to chemicals, dusts, fumes, and the level of preventive measures used in occupational settings e.g., ventilation, and use of personal protective equipment. The assessment of occupational exposure to unintentionally released nanoparticles of each patient was independently and anonymously performed by an experienced industrial hygienist, follow-up on the review of occupational physicians, on the basis of these data. A probability of exposure to unintentionally produced nanoparticles from work-processes implemented in each occupation held by the patients was determined. Classes of probability were defined in a four scale: 0 when it was not found, 1 when it was possible (<10%), 2 when it was likely (10–50%), and 3 when it was very likely (>50%). Then they considered the final probability of exposure to unintentionally released nanoparticles as the highest probability of exposure observed in the career.

## 3. Results

### 3.1. Relationship between Biomonitoring of Nanoparticles in Broncho-Alveolar Lavages and Lung Diseases

We previously separated micro from submicron and nanoparticles contained in BAL and BW samples from 100 patients suffering from ILD. We then assessed the metal load in each of these fractions. Results are fully detailed in our previous publications [18,19].

**Figure 2:**
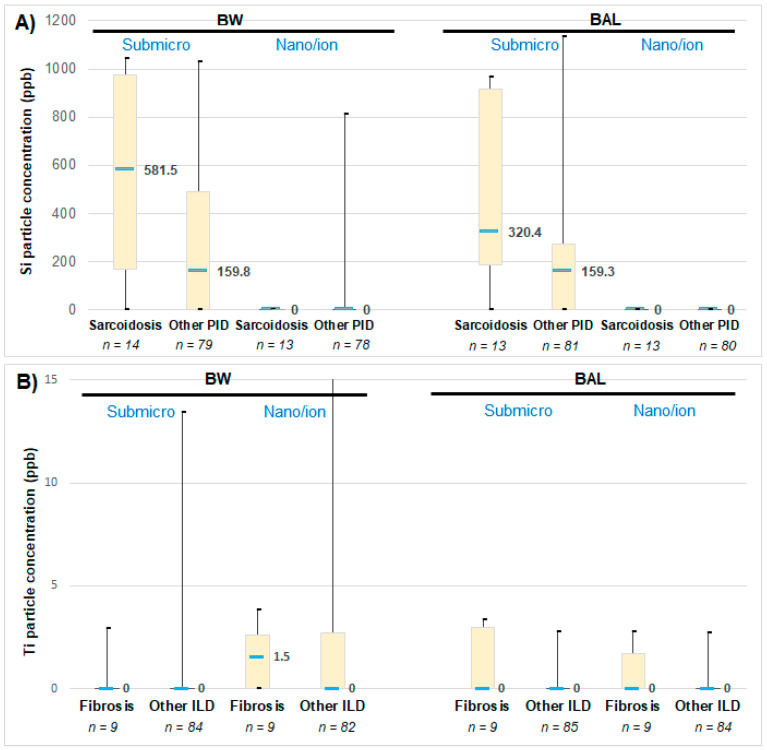
(**A**) Si particles concentration in bronchial wash (BW) and broncho-alveolar lavages (BAL) of patients suffering either from sarcoidosis or another type of ILD. (**B**) Ti particles concentration in BW and BAL of patients suffering either from idiopathic pulmonary fibrosis or another type of ILD. Comparison between the fractions containing either the submicron particles and the nanoparticles and ions. The median (in bold), minimal and maximal values, as well as the first and third quartiles are indicated. The number of patients (*n*) from each group is reported (please note that some data are missing because for technical reasons some BW and BAL samples could not be analyzed).

As shown by Figure 2A, we evidenced a concentration of submicron silica particles higher in patients suffering from sarcoidosis than in patients suffering from other ILD. Similarly, we observed a concentration of titanium nanoparticles higher in patients suffering from idiopathic fibrosis than in patients suffering from other ILD (Figure 2B).

We further analyzed potential relationships between the particle load in BAL and BW and the patients’ gender and their past or current smoking status by calculating the Pearson correlation coefficient. No correlation was observed (data not shown).

### 3.2. Relationship between Lung Diseases and Occupational Exposure

To explore possible associations between interstitial lung diseases and occupational exposure to airborne nanoparticles, we considered the highest probability of exposure to unintentionally released nanoparticles observed in the career. Results are reported Table 2.

**Figure 3:**
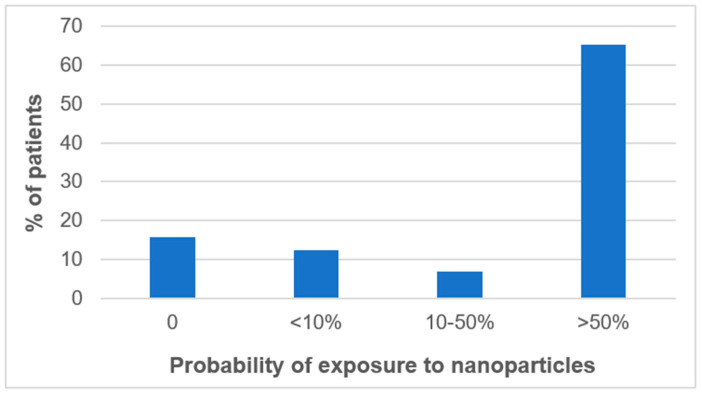
Distribution of the patients depending on their probability of exposure to unintentionally released nanoparticles.

Figure 3 reports the distribution of the patients, irrespective of the disease they suffer from, depending on their final probability of exposure to unintentionally released nanoparticles.

We first observed that few patients (16%) had a null probability of exposure to unintentionally released nanoparticles during their occupational life. On the contrary, the vast majority of the patients (65%) exhibited a high probability of exposure to unintentionally released nanoparticles (>50%).

**Figure 4:**
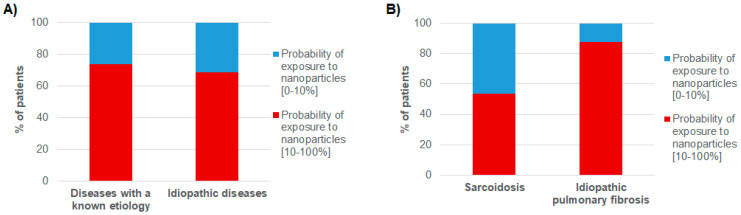
(**A**) Distribution of patients depending on the probability of exposure to unintentionally released nanoparticles and depending on the origin of their disease. (**B**) Distribution of patients depending on the probability of exposure to unintentionally released nanoparticles and depending on the nature of their disease.

As shown by Figure 4A, we then reported the distribution of patients depending on their probability of exposure to unintentionally released nanoparticles (we grouped the 0–10% probability of exposure to unintentionally released nanoparticles on one hand and the 10–100% probability on the other hand) and the origin of their disease (either with a known etiology or idiopathic).

The probability of exposure to unintentionally released nanoparticles was higher than 10% for a large majority of patients, either suffering from a disease with a known etiology or from an idiopathic disease (74% and 69% respectively).

We then focused our attention on sarcoidosis and idiopathic pulmonary fibrosis, two idiopathic diseases for which our mineralogical analyses had suggested correlations with the concentration of submicron silica particles and that of titanium nanoparticles, respectively (Figure 4B).

Interestingly, we observed different profiles between the two types of diseases. Regarding sarcoidosis, patients were almost equally distributed between the group of low probability of exposure to unintentionally released nanoparticles and that with a probability of exposure higher than 10%. On the contrary, for idiopathic pulmonary fibrosis, the probability of exposure to unintentionally released nanoparticles was higher than 10% for almost 88% of the patients.

## 4. Discussion

Besides the widely used in vivo and in vitro studies, mineralogical analyses of human biological samples can bring interesting and useful information, especially to investigate relationship between exposure to airborne nanoparticles and idiopathic lung diseases. For these reasons, here we go one step further and couple biomonitoring to exposure estimates based on expert judgments. Thus, we underwent a retrospective occupational exposure to unintentionally emitted nanoparticles assessment. This was mainly based on expert’s judgment through job title, workplace conditions and their knowledge of occupations and similar documented situations, that may however lead to possible sources of errors in the estimates. We thus observed that most of the patients, whatever the type of disease they suffer from, showed a high probability of exposure to unintentionally released nanoparticles (Figure 3). This observation could suggest a potential contribution of inhaled nanoparticles to the development or exacerbation of lung diseases. This is particularly true for idiopathic pulmonary fibrosis where 88% of the patients exhibited a high probability of exposure to unintentionally released nanoparticles (Figure 4B). This finding is consistent with our mineralogical analyses that suggested a role of titanium nanoparticles in this disease. However, regarding sarcoidosis, only half (54%) of the patients were classified in the group of high probability of exposure to unintentionally released nanoparticles. Once again, this observation is in agreement with our mineralogical analyses that previously highlighted a potential contribution of silica submicron particles, i.e., particles bigger than nanoparticles, in this disease [19].

To the best of our knowledge, only two studies have established a clear relationship between exposure to unintentionally released nanoparticles and long-term negative effects in humans. Song et al. [24,25] found silica nanoparticles in clinical samples from seven patients suffering from lung injuries after an occupational exposure. However, these patients were also exposed to other toxic substances; consequently, no firm conclusion could be reached. Another study [26] had reported that pulmonary injuries were more severe in welders than in unexposed people suggesting that nanoparticles present in welding fumes could be responsible, at least in part, for the pulmonary inflammation. But this study was limited to the description of few clinical cases and did not have a significant statistical power. It was also restricted to a target population and conclusions can hardly be extrapolated.

In the literature, the investigation of inhaled nanoparticles’ presence in patients’ lungs is rare and when it exists it is limited to electron microscopy observations that do not allow a complete physicochemical characterization of the nanoparticles and is not suitable for large cohort analysis as it is a time-consuming and expensive technique. Laser-induced breakdown spectroscopy (LIBS) could appear as a promising alternative for the direct visualization of endogenous or exogenous elements within tissues but is still under development and not routinely used for biomedical applications [27]. Nevertheless, the biomonitoring of nanoparticles in biological samples appears as a promising approach to get new insights into the understanding of the genesis or evolution of lung diseases due to nanoparticle exposure.

In the present paper, we propose to couple biomonitoring to the assessment of unintentionally released nanoparticles exposure to get a larger picture on the relationship between exposure to airborne nanoparticles and interstitial lung diseases. Although this strategy has several advantages as previously discussed, it has also some limitations we have to take into account. First, results should be considered with caution as we have a small number of patients, especially in the idiopathic pulmonary disease group (nine patients). Moreover, no significant difference between the unintentionally released nanoparticle exposure assessment of different distinguished interstitial lung diseases groups was observed (results not shown). As we said above, further investigations are necessary to confirm the results observed. We may also remind the reader that we performed our analyses in a cohort of patients. It should be interesting to compare these data to those obtained with healthy control subjects. However, for ethical reasons, it is impossible to perform broncho-alveolar lavages in healthy persons due to the invasive nature of this exam. We should mention that we focused our analyses on occupational exposure to unintentionally released nanoparticles, to be complete, we should also consider other sources of exposure to nanoparticles, for instance environmental exposure (taking into account patients’ living area, mode of transport, of heating, leisure or use of hygiene and cosmetics products…). However, these data are much more complex to collect with accuracy. Finally, it should be kept in mind that the presence of a given particle in a larger amount within biological samples is not sufficient to prove a causal link with a disease, and toxicity assessment is necessary to demonstrate a pathogenic effect as well as mechanistic studies to understand the underlying mechanisms. One perspective we propose to strengthen our array of presumptions is to couple the nanoparticle biomonitoring in lung clinical samples to the in vitro assessment of their toxicity [14]. For instance, by incubating cells with nanoparticles extracted from patients’ BAL and then analyzing the cell response in terms of induction of cell death, pro-inflammatory response, oxidative stress, etc. The advantage of such strategy is that the in vitro assays are performed using nanoparticles which nature and dose are representative of real-life. From such approach benefits could be expected in the therapeutic and/or prevention fields. Indeed, the knowledge of the toxicity potential of nanoparticles can lead to preventive measures to limit the exposure to the harmful substances.

We determined a probability of exposure to unintentionally produced nanoparticles present in polluting emissions during each occupation held by the patients. We did not determine intensity and frequency of nanoparticle exposure. We also observed that the probability of exposure alone was not useful to discriminate some exposure differences between different distinguished interstitial lung diseases groups. Furthermore, our results did not determine the extent of occupational or daily life exposure and recent or lifetime exposure to unintentionally released nanoparticles. However, given the current state of knowledge on occupational exposure levels, which is patchy and based on heterogeneous methods, we have chosen to limit the assessment to probability [28]. The nanoparticle assessment used in this study was possible thanks to the knowledge acquired in the framework of the development of the job-exposure matrix MatPUF [29]. Such a job-exposure matrix might be useful to improve the quality and the accuracy of nanoparticle exposure assessment during a full occupational career, providing for each job chronological exposure data and main chemical families of released nanoparticles. MatPUF job-exposure matrix has already been used in epidemiological studies and has made it possible to highlight a relationship between occupational exposure to unintentionally released nanoparticles and small for gestational age and, cancers, such as lung cancer and brain nervous system tumors [30,31].

Job-exposure matrices have already been used to investigate associations between occupational exposure and idiopathic pulmonary fibrosis, however conflicting results were reported. Indeed, while Abramson et al. showed that occupational exposures to specific organic, mineral, or metal dusts were not associated with idiopathic pulmonary fibrosis [32], on the contrary Andersson et al. reported that occupational exposure to inorganic dusts, excluding silica and asbestos, was associated with increased risk of idiopathic pulmonary fibrosis [33]. Regarding sarcoidosis, both Graff et al. and Jonsson et al. concluded that occupational exposure to silica dust led to an increased risk of sarcoidosis [34,35]. These studies confirmed that lung diseases such as idiopathic pulmonary fibrosis or sarcoidosis could be caused by the inhalation of mineral particles [36,37,38]. Thus, such job-exposure matrix are interesting and useful tools for the assessment of occupational UFP exposure that can both contribute to the improvement of epidemiological knowledge of health risks and to the implementation of prevention in the workplace.

Nanoparticles possess nanostructure-dependent properties (e.g., chemical, physical, biological), which make them desirable for commercial or industrial applications. Workers are increasingly exposed to nanoparticles in occupational settings. However, these same properties may potentially lead to atypical toxicity and health risks are yet unknown. For these reasons, there is an unceasing scientific interest to the human toxicokinetics and toxicodynamics of nanoparticles and concerns about the potential risks of exposure to humans have been raised. Although toxic effects have not been really demonstrated in humans, there is accumulating evidence from experimental studies that exposure to some nanoparticles may be harmful [39]. However, it is mostly based on in vitro tests and animal experiments. Key questions, regarding the duration and level of exposure in humans, the toxic behavior of nanoparticles in humans, the physiological and chemical interaction with human body, the harmlessness of these interactions, and acute or chronic effects adverse effects need to be resolved [40]. As potential occupational exposure to unintentionally released nanoparticles becomes more prevalent, it is important that the principles of risk assessment and risk management be considered for workers. The risk assessment/risk management framework comprises three essential components: research, risk assessment, and risk management [40]. This exploratory study had the objective to characterize exposure to unintentionally released nanoparticles, using association of two approaches, biomonitoring and occupational exposure to unintentionally released nanoparticles assessment, in patients with ILD, to study possible links between with these health outcomes and exposure to unintentionally released nanoparticles and, thus, to contribute to the knowledge to risk factors of occupational and environmental lung ILD. Moreover, these approaches might be used to contribute to nanoparticles risk assessment. In fact, quantification of unintentionally released nanoparticles exposure remains the major challenge for prevention.

## 5. Conclusions

Large epidemiological studies are too personnel, require financial resources, and are time-consuming. By combining mineralogical analyses of human BAL samples and estimation of occupational nanoparticle exposure, the relationship between occupational exposure to airborne nanoparticles, nanoparticle lung burden, and implications on the etiology of lung diseases can be investigated. These complementary approaches appear as a promising strategy to get a comprehensive picture and could bring informative data for human health risk assessment and management.

Following this approach, it is evidenced that most of the patients from our cohort, whatever the type of disease they suffer from, showed a high probability of exposure to unintentionally released nanoparticles. This observation was consistent with the nanoparticle lung burden previously assessed, suggesting a potential role of inhaled nanoparticles to the development or exacerbation of lung diseases, although further experiments are necessary to draw firm conclusions.

## Figures and Tables

**Table 1:** Description of the cohort.

Disease	Number of Patients	Median Age (Min–Max)	Sex Ratio (M:F)	Smokers (Former Smokers)
**With a known etiology**	**58**	**70.5 (22–87)**	**43:15**	**10.3% (46.6%)**
Drug related ILD	15	71 (46–81)	14:1	6.7% (73.3%)
Infectious ILD	12	69.5 (42–85)	7:5	16.7% (33.3%)
Hypersensitivity pneumonitis	7	75 (34–78)	6:1	0% (14.3%)
Auto-immune pneumonitis	5	72 (22–87)	2:3	0% (40%)
Lymphangitis carcinomatosis/Neoplasia	4	75.5 (67–83)	2:2	0% (50%)
Desquamative interstitial pneumonia	3	71 (48–81)	2:1	66.7% (0%)
Pneumoconiosis	2	50 (41–59)	2:0	50% (50%)
Pulmonary veno-occlusive disease	2	73 (72–74)	2:0	0% (100%)
Antisynthetase syndrome	2	70.5 (70–71)	1:1	0% (50%)
Silicosis	1	55	1:0	0% (100%)
Microscopic polyangiitis	1	71	0:1	0% (0%)
Granulomatosis with polyangitis (Wegener’s granulomatosis)	1	65	1:0	0% (0%)
Left heart failure	1	59	1:0	0% (100%)
Lipoid pneumonia	1	69	1:0	0% (100%)
Bronchiolitis obliterans	1	40	1:0	0% (0%)
**Idiopathic**	**34**	**67.5 (25–81)**	**22:12**	**14.7% (23.5%)**
Sarcoidosis	14	47 (25–80)	6:8	14.3% (7.1%)
Idiopathic nonspecific interstitial pneumonia	11	76 (46–81)	8:3	9.1% (27.3%)
Idiopathic pulmonary fibrosis	9	69 (61–81)	8:1	22.2% (36.4%)
**Others**	**8**	**61 (46–83)**	**3:5**	**0% (28.6%)**

**Table 2:** Description of the patients’ cohort in terms of lung disease, occupations and probability of exposure to unintentionally released nanoparticles. The probability of exposure assessment resulted from the nanoparticle-release potential of the considered work process (determined from the literature) which was adjusted for each occupation according to the description of tasks and work-processes implemented as provided in the ISCO 1968 classification.

**Patient Number**	**Lung Disease**	**Group (E: Disease of Known Etiology, I: Idiopathic Disease)**	**Occupations**			**Probability of Exposure to Nanoparticles: 0 Not Found, 1: Possible < 10%, 2: Likely 10–50%, 3: Very Likely > 50%**			**Final Exposure to Nanoparticles Probability: Highest Probability of Exposure to Nanoparticles in the Career**
			1	2	3	Occupation 1	Occupation 2	Occupation 3	
1	Drug related ILD	E	Coachbuilder/painter	Welder	Printing machine operator	3	3	1	**3**
2	Idiopathic pulmonary fibrosis	I	Textile products machine operator			1			**1**
3	Drug related ILD	E	Farmer	Switching operator	Switching operator	3	1	1	**3**
4	Lymphangitis carcinomatosis/Neoplasia	E	Farmer			2			**2**
5	Other		Mason	Refractory bricklayer	Ceramics operator	3	3	3	**3**
6	Drug related ILD	E	Market gardener	Farmer	Farm hands	0	2	1	**3**
7	Other		Seamstress	Cook	Childminder	1	3	1	**3**
8	Other		Floor sander	Truck driver	Pressman	3	3	1	**3**
9	Idiopathic pulmonary fibrosis	I	Miner	Miner	Train driver	3	3	3	**3**
10	Drug related ILD	E	Miner	Tile setter		3	3		**3**
11	Auto-immune pneumonitis	E	Domestic help	Domestic help		1	1		**1**
12	Drug related ILD	E	Farmer			3			**3**
13	Hypersensitivity pneumonitis	E	Bank employee			0			**0**
14	Infectious ILD	E	Accountant	Medical secretary		0	0		**0**
15	Other		/						
16	Infectious ILD	E	Printing machine operator	Printing machine operator		1	1		**1**
17	Lymphangitis carcinomatosis/Neoplasia	E	Masseuse	Chocolate—products machine operator	Waitress/manageress	0	0	1	**1**
18	Infectious ILD	E	Teacher	Teacher		0	0		**0**
19	Idiopathic pulmonary fibrosis	I	Mason			3			**3**
20	Drug related ILD	E	Truck driver	Salesman	Company director	3	0	0	**3**
21	Drug related ILD	E	Pipe fitter/welder			3			**3**
22	Desquamative interstitial pneumonia	E	Coachbuilder/painter			3			**3**
23	Drug related ILD	E	Waitress	Waitress	Candle production machine operator	0	2	0	**2**
24	Other		Factory worker	Factory worker	Market gardener	0	3	0	**3**
25	Pulmonary veno-occlusive disease	E	Farmer			3			**3**
26	Idiopathic pulmonary fibrosis	I	Printing machine operator	Truck driver	Salesman	1	3	0	**3**
27	Idiopathic nonspecific interstitial pneumonia	I	Mason	Joiner		3	3		**3**
28	Idiopathic nonspecific interstitial pneumonia	I	Secretary	Secretary	Domestic help	0	0	1	**1**
29	Drug related ILD	E	Mason	Factory worker		3	1		**3**
30	Sarcoidosis	I	Seamstress	Childminder	Seamstress	1	0	0	**1**
31	Drug related ILD	E	Baker	Post officer	Foundry worker	3	0	3	**3**
32	Pneumoconiosis	E	Dental prosthetist			3			**3**
33	Auto-immune pneumonitis	E	Carer			0			**0**
34	Idiopathic nonspecific interstitial pneumonia	I	Baker			3			**3**
35	Bronchiolitis obliterans	E	Boilermaker/welder	Pipe fitter/boilermaker/welder		3	3		**3**
36	Other		Cleaner	Saleswoman	Waitress/manageress	0	0	1	**1**
37	Idiopathic nonspecific interstitial pneumonia	I	Domestic help	Food salesperson	Cashier	1	2	0	**2**
38	Sarcoidosis	I	Farmer			3			**3**
39	Antisynthetase syndrome	E							
40	Pulmonary veno-occlusive disease	E	Farmer			3			**3**
41	Idiopathic pulmonary fibrosis	I	Plant operator	Plant operator	Cleaner	1	3	1	**3**
42	Granulomatosis with polyangitis (Wegener’s granulomatosis)	E	Joiner			3			**3**
43	Sarcoidosis	I	Seamstress	Cleaner	Childminder	1	1	0	**1**
44	Left heart failure	E	Boilermaker/welder	Carpenter/metal fitter	Carpenter/metal fitter	3	3	3	**3**
45	Hypersensitivity pneumonitis	E	Farmer			3			**3**
46	Infectious ILD	E	Joiner			3			**3**
47	Idiopathic pulmonary fibrosis	I	Manufacturing labourer	Manufacturing labourer		3	3		**3**
48	Sarcoidosis	I	Salesman	Accountant		0	0		**0**
49	Idiopathic nonspecific interstitial pneumonia	I	Butcher			1			**1**
50	Idiopathic pulmonary fibrosis	I	Manufacturing labourer	Baker	Mason	3	3	3	**3**
51	Pneumoconiosis	E	Joiner			3			**3**
52	Microscopic polyangiitis	E	Saleswoman			0			**0**
53	Idiopathic nonspecific interstitial pneumonia	I	Machine-tool operator	Manufacturing labourer	Fiberglass plant operator	3	1	1	**3**
54	Desquamative interstitial pneumonia	E	Waitress	Fruit picker	Cleaner in a plastic products factory	2	0	2	**2**
55	Sarcoidosis	I	Animator in retirement home			0			**0**
56	Idiopathic nonspecific interstitial pneumonia	I	Foundry moulder			3			**3**
57	Idiopathic pulmonary fibrosis	I	Hospital caregiver			2			**2**
58	Sarcoidosis	I	Cleaner			2			**2**
59	Idiopathic pulmonary fibrosis	I							
60	Other		Speech therapist	Speech therapist	Speech therapist	0	0	0	**0**
61	Infectious ILD	E	Gym teacher			0			**0**
62	Auto-immune pneumonitis	E	Farmer	Textile products machine operator		3	1	0	**3**
63	Drug related ILD	E	Optical assembler	Butcher		0	0		**0**
64	Lipoid pneumonia	E	Steel materials handling	Machine-tool operator	Mason	3	3	3	**3**
65	Desquamative interstitial pneumonia	E	Metal carpenter			3			**3**
66	Sarcoidosis	I	Secretary	Policeman		0	0		**0**
67	Auto-immune pneumonitis	E	Office worker	Communications manager	Director	1	0	1	**1**
68	Infectious ILD	E	Plasterer/painter	Handler		3	2		**3**
69	Hypersensitivity pneumonitis	E	Cleaner	Quality manager	Windshield manufacturer	2	0	3	**3**
70	Auto-immune pneumonitis	E	Joiner			3			**3**
71	Other								
72	Infectious ILD	E	Carer			0			**0**
73	Hypersensitivity pneumonitis	E	Wood-products machine operator	Farmer		3	3		**3**
74	Drug related ILD	E	Metal polish			3			**3**
75	Drug related ILD	E	Baker	Salesman	Estate agent	3	1	1	**3**
76	Hypersensitivity pneumonitis	E	Mason	Train driver		3	3		**3**
77	Sarcoidosis	I	Manufacturing labourers	Construction sites truck driver	Manufacturing labourers	3	3	3	**3**
78	Infectious ILD	E	Mason	Rubber products (tyre) machine operator	Textile products machine operator	3	3	1	**3**
79	Drug related ILD	E	Metal industry operator	Machine-tool operator	Boilermaker	3	3	3	**3**
80	Idiopathic nonspecific interstitial pneumonia	I	Manufacturing labourers	Textile products machine operator		3	1		**3**
81	Sarcoidosis	I							
82	Lymphangitis carcinomatosis/Neoplasia	E	Meter reader	Storekeeper	Executive	0	0	0	**0**
83	Idiopathic nonspecific interstitial pneumonia	I	Textile products machine operator			1			**1**
84	Antisynthetase syndrome	E	Baker	Machine-tool operator	Security officer	3	3	0	**3**
85	Infectious ILD	E	Farmer			3			**3**
86	Silicosis	E	Miner	Mason		3	3		**3**
87	Sarcoidosis	I	Mason			3			**3**
88	Infectious ILD	E	Textile products machine operator			3			**3**
89	Infectious ILD	E	Chocolate—products machine operator			1			**1**
90	Hypersensitivity pneumonitis	E	Plumber			3			**3**
91	Drug related ILD	E	Machine-tool operator	Machine-tool operator	Metal coating machine operator	3	3	3	**3**
92	Idiopathic nonspecific interstitial pneumonia	I	Boilermaker			3			**3**
93	Sarcoidosis	I	Electrician	Electrician in food industry	Electrician in mining plant	1	1	3	**3**
94	Idiopathic nonspecific interstitial pneumonia	I	Farmer	Machine finishing	Machine operator	3	3	2	**3**
95	Infectious ILD	E	Post officer			0			**0**
96	Lymphangitis carcinomatosis/Neoplasia	E	Machine-tool operator	Policeman		3	0		**3**
97	Sarcoidosis	I	Train controller	Music teacher		0	0		**0**
98	Hypersensitivity pneumonitis	E	Welder/machine-tool operator			3			**3**
99	Sarcoidosis	I	Electrician			3			**3**
100	Sarcoidosis	I	Mason	Mover	Metal-heat-treating plant operator	3	1	3	**3**
